# Perioperative Management of Hereditary Antithrombin Deficiency in a Patient Undergoing Minimally Invasive Thoracic Surgery: A Case Report

**DOI:** 10.70352/scrj.cr.25-0166

**Published:** 2025-12-18

**Authors:** Hiromitsu Domen, Yuka Takakuwa, Hidehisa Yamada

**Affiliations:** Department of Thoracic Surgery, NTT Medical Center Sapporo, Sapporo, Hokkaido, Japan

**Keywords:** uniportal robotic-assisted thoracic surgery, hereditary antithrombin deficiency, anterior mediastinal tumor, perioperative management

## Abstract

**INTRODUCTION:**

Hereditary antithrombin deficiency is a rare congenital disorder associated with an increased risk of venous thromboembolism, particularly during high-risk situations such as surgery. Effective perioperative anticoagulation management is critical to prevent thromboembolic complications in these patients.

**CASE PRESENTATION:**

A 55-year-old female with a history of deep vein thrombosis and hereditary antithrombin deficiency presented with an anterior mediastinal tumor. Imaging findings suggested a benign cystic lesion; however, malignancy could not be completely excluded. A perioperative management strategy involving recombinant human antithrombin and heparin therapy was employed to safely perform uniportal robotic-assisted thymic cyst resection via a 4-cm incision in the right 5th intercostal space. Postoperative assessment, including clinical monitoring and follow-up imaging, confirmed the absence of thromboembolic complications. Histopathological examination revealed a benign thymic cyst.

**CONCLUSIONS:**

This case highlights the importance of individualized perioperative anticoagulation management in patients with hereditary antithrombin deficiency undergoing thoracic surgery. The combination of recombinant human antithrombin and heparin therapy provided effective anticoagulation, allowing successful surgical intervention without thromboembolic events. Establishing standardized protocols for the management of such high-risk patients is essential for improving surgical safety and outcomes.

## Abbreviations

AT
: antithrombin
APTT
: activated partial thromboplastin time
DOACs
: direct oral anticoagulants
FDG-PET
: 18F-fluorodeoxyglucose positron emission tomography
RATS
: robotic-assisted thoracic surgery
rhAT
: recombinant human antithrombin
U-RATS
: uniportal robotic-assisted thoracic surgery
U-VATS
: uniportal video-assisted thoracic surgery
VTE
: venous thromboembolism

## INTRODUCTION

Hereditary AT deficiency is a rare congenital disorder characterized by a deficiency or dysfunction of AT, a critical inhibitor of thrombin and factor Xa. This condition predisposes individuals to VTE, particularly during situations that increase the risk of thrombosis, such as surgery, trauma, pregnancy, or prolonged immobility. Surgical procedures in patients with hereditary AT deficiency require specialized perioperative management to balance the risk of thromboembolism against the risk of bleeding due to anticoagulation therapy.^1)^

Minimally invasive RATS has been increasingly utilized in thoracic surgery due to its advantages in precision, dexterity, and visualization compared with conventional thoracoscopic approaches. Among the various RATS techniques, U-RATS has emerged as a novel, minimally invasive approach designed to reduce surgical trauma, enhance cosmetic outcomes, and improve postoperative recovery. Studies have suggested that U-RATS may offer advantages over conventional multiport RATS and U-VATS in terms of shorter hospital stays, reduced postoperative pain, and better cosmetic results.^2–5)^ However, high-level evidence comparing U-RATS to other techniques remains limited, and its application in patients with coagulation disorders has not been well-documented.

This case report describes the perioperative management of a patient with hereditary AT deficiency who underwent minimally invasive thoracic surgery for an anterior mediastinal tumor. The focus of this report is the careful anticoagulation management strategy employed to ensure a safe surgical procedure, as well as the feasibility of utilizing U-RATS as a minimally invasive approach in this unique patient population.

## CASE PRESENTATION

A 55-year-old female was referred to our hospital after an incidental finding of an abnormal shadow on a chest X-ray during a routine medical checkup. At the age of 20, she had experienced a pulmonary embolism after developing blood clots in both lower extremities. She was treated with an inferior vena cava filter and placed on warfarin. A blood sample taken at that time revealed AT deficiency, and a family history of thrombosis led to the diagnosis of congenital AT deficiency. Warfarin was switched to apixaban at the age of 53.

**Fig. 1:**
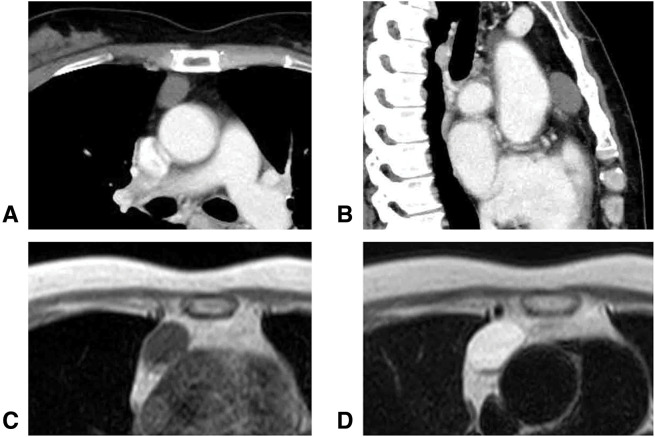
Preoperative imaging. Chest CT shows a 28 × 15-mm round nodule in the anterior mediastinum (**A**, **B**). MRI shows the nodule as low intensity on T1-weighted imaging (**C**) and high intensity on T2-weighted imaging (**D**).

A chest CT scan revealed a 28 × 15-mm round nodule in the anterior mediastinum (**Fig. 1A** and **1B**). MRI showed the nodule to be of low intensity on T1-weighted images (**Fig. 1C**) and high intensity on T2-weighted images (**Fig. 1D**). No hyperaccumulation was noted on FDG-PET.

The differential diagnosis based on these imaging findings included thymic cyst, pericardial cyst, benign teratoma, and early-stage thymoma. While the imaging characteristics were suggestive of a benign lesion, the possibility of malignancy could not be completely excluded. The patient strongly desired surgical resection due to her anxiety about potential malignancy and the need for a definitive diagnosis. Taking into account the patient’s background of hereditary AT deficiency, we planned the surgery carefully with a comprehensive perioperative anticoagulation strategy.

### Perioperative management

**Fig. 2:**
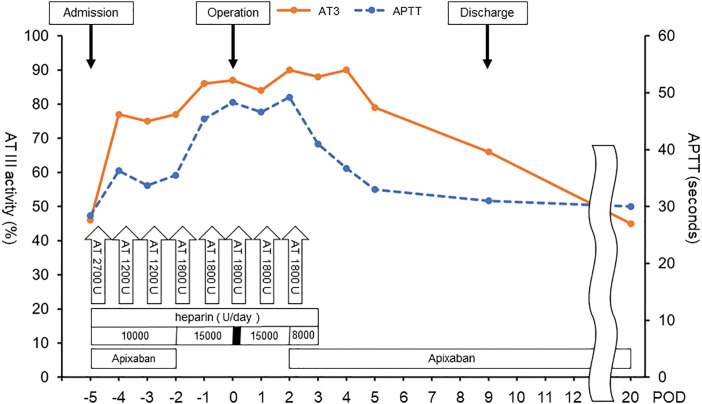
Perioperative management. The patient was admitted 5 days before surgery. Recombinant human antithrombin and heparin were initiated on the day of admission. Apixaban was discontinued 2 days before surgery. Heparin was stopped 4 hours prior to surgery and resumed immediately postoperatively. The thoracic drain was removed on POD 1. Apixaban was resumed on POD 2, and rhAT and heparin were stopped on POD 3. There was no evidence of thrombus formation throughout the procedure. AT, antithrombin; APTT, activated partial thromboplastin time; rhAT, recombinant human antithrombin

Details of blood tests and medications administered during the perioperative period are shown in **Fig. 2**. The patient was admitted to the hospital 5 days before surgery. Preoperative blood tests showed an AT activity of 46% and an APTT of 28.4 seconds. rhAT (ACOALAN Injection; Japan Blood Products Organization, Tokyo, Japan) and heparin were started upon admission, as recommended based on previous guidelines and reports.^1,6)^ Heparin was given at a dose of 10000 units/day with an APTT target of 30–40 seconds while apixaban was discontinued 2 days prior to surgery. The heparin dosage was adjusted to maintain an APTT of around 50 seconds. Heparin was stopped 4 hours before surgery, and resumed immediately after the operation. The total withdrawal time was approximately 6 hours.

Following the procedure, anticoagulation management was carefully adjusted. Heparin was resumed immediately after surgery and was continued throughout the perioperative period, including during chest drain removal on POD 1. Blood tests performed on the morning of POD 1, prior to chest drain removal, showed an APTT of 46.6 seconds, indicating mild prolongation. This degree of prolongation was considered acceptable, and the risk of bleeding complications related to chest drain removal was deemed low. After successful removal of the drain, heparin administration was continued until it was discontinued on POD 3. Apixaban was restarted on POD 2. On POD 5, AT III activity was measured at 79% and APTT at 33 seconds. The patient was discharged on POD 9. During outpatient follow-up on POD 20, AT III activity was measured at 45% and APTT at 30 seconds.

Regarding the assessment of thrombus formation, our approach was based on clinical observation and the absence of symptoms suggestive of thromboembolism. Similar approaches of perioperative AT replacement therapy have been previously reported as effective in cardiac surgeries involving AT-deficient patients.^6)^ The patient had a history of deep vein thrombosis and pulmonary embolism around the age of 20, presenting with clear clinical symptoms such as leg swelling, dyspnea, and respiratory distress. During the perioperative period, we carefully monitored for similar symptoms, which did not occur at any point.

Contrast-enhanced CT imaging was performed 6 months postoperatively, and no evidence of thrombus formation was observed. Although we did not perform early postoperative imaging studies to detect thrombi, the absence of any clinical symptoms suggested the absence of thrombus formation. This approach was based on the patient’s previous presentation and ongoing clinical monitoring.

Histopathologically, a 3.2-cm cystic mass was found, the inner lumen of which was lined with ciliated columnar epithelium and pseudostratified ciliated epithelium, but the cells that constituted it showed little atypia, and the tumor was diagnosed as a benign thymic cyst.

### Operation

**Fig. 3:**
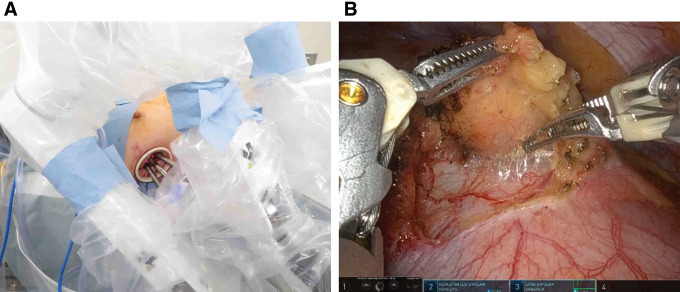
Operation. (**A**) A 4.0-cm skin incision was made at the right 5th intercostal space along the midaxillary line to access the thoracic cavity. The Da Vinci Xi (Intuitive Surgical, Sunnyvale, California, USA) robot was positioned, and Arm 4 was moved caudally. An 8-mm, 30° oblique endoscope was attached to Arm 1, a fenestrated bipolar forceps to Arm 2, and a long bipolar grasper to Arm 3. The depths of the ports were adjusted to prevent interference. (**B**) The lesion, identified as a soft induration with slight elevation in the right anterior mediastinum, was excised via partial thymectomy.

The procedure was performed using the U-RATS technique. The patient was positioned in the supine position under general anesthesia. A 4.0-cm skin incision was made at the right 5th intercostal space along the midaxillary line to access the thoracic cavity (**Fig. 3A**).The lesion, identified as a soft induration with slight elevation in the right anterior mediastinum, was excised via partial thymectomy (**Fig. 3B**). Console time was 32 minutes, total operative time was 1 hour 6 minutes, and there was no blood loss.

A **Supplementary Video 1** demonstrating the key steps of the U-RATS, including port placement, instrument manipulation, and lesion resection, is provided for enhanced comprehension of the surgical procedure.

## DISCUSSION

AT deficiency is a rare autosomal dominant disorder characterized by a reduced ability to inhibit thrombin and other activated coagulation factors, resulting in a hypercoagulable state. The prevalence of hereditary AT deficiency in the general population is estimated to be between 0.02% and 0.17%.^6,7)^ There are 2 main types: Type I, which is a quantitative deficiency with reduced AT levels and activity, and Type II, a qualitative deficiency with normal levels but impaired function due to mutations affecting specific sites.^6)^

Historically, perioperative management of patients with AT deficiency has relied on achieving adequate AT activity to prevent thromboembolic events, particularly during high-risk situations such as surgery and childbirth. Achieving preoperative AT activity of ≥120% and maintaining postoperative levels at ≥80% is essential for reducing the risk of complications, as demonstrated by Nishimura and Takagi, who successfully managed 5 patients undergoing cardiovascular surgery using AT concentrate.^6)^ Similarly, Kitahara et al. reported successful perioperative management of a right atrial hemangioma in a patient with AT deficiency by maintaining AT activity at 80% using AT concentrate.^7)^

In recent years, rhAT has emerged as a safer alternative to human plasma-derived AT due to the elimination of infection risks associated with blood-derived products. Fujibe et al. demonstrated the effective use of rhAT during labor in a pregnant female with hereditary AT deficiency, achieving AT activity of 80%–120% and preventing thromboembolism without adverse effects.^8)^ This supports the potential role of rhAT as a valuable option for patients in whom infection risk or unpredictable availability of hpAT is a concern.

Additionally, DOACs such as rivaroxaban or apixaban have been explored as alternative anticoagulation strategies. Kawai et al. reported a successful case of perioperative management using rivaroxaban in an elderly patient with AT deficiency, suggesting that DOACs may be suitable for high-risk patients who cannot receive conventional anticoagulant therapy.^9)^

These findings underscore the importance of individualized perioperative management for patients with AT deficiency. The combination of traditional AT concentrate administration, emerging alternatives such as rhAT, newer anticoagulant options like DOACs may contribute to safer and more effective surgical outcomes.

In this case, our approach was based on previously reported guidelines and case reports. rhAT was administered preoperatively to raise AT activity above the desired level, while intravenous heparin was initiated and adjusted to achieve an APTT of approximately 50 seconds.^9)^ Apixaban was discontinued 2 days before surgery and resumed on POD 2, with heparin administration continuing until rhAT was deemed unnecessary. The decision to proceed with surgery was made after careful consideration of the potential risk of malignancy and the patient’s strong desire for definitive diagnosis and treatment. Postoperative contrast-enhanced CT confirmed the absence of thrombus formation, and anticoagulation therapy was successfully managed throughout the perioperative period.

The use of U-RATS was considered advantageous due to its potential for minimizing surgical trauma, reducing postoperative pain, and improving cosmetic outcomes compared with conventional approaches. While high-level evidence comparing U-RATS to multiport RATS and U-VATS remains limited, previous reports have suggested that U-RATS can be safely performed with acceptable perioperative outcomes.^3–5,10)^ The enhanced precision and dexterity of robotic instruments may provide better control during dissection, particularly in challenging cases involving coagulation disorders. However, the decision to utilize U-RATS in this case was not solely based on its theoretical advantages but also on our institutional experience and the availability of the robotic system.

Although our management approach was successful, it is important to recognize the limitations of this report. This is a single case report, and further studies are needed to establish standardized guidelines for perioperative management of patients with hereditary AT deficiency undergoing thoracic surgery. Additionally, while U-RATS may offer potential advantages in terms of minimal invasiveness, its safety and efficacy in patients with coagulation disorders require further investigation.

## CONCLUSIONS

This case highlights the importance of meticulous perioperative anticoagulation management in patients with hereditary AT deficiency undergoing minimally invasive thoracic surgery. The successful application of rhAT and heparin therapy allowed for safe surgical resection without thromboembolic complications. Additionally, the use of U-RATS demonstrated its feasibility as a minimally invasive approach, offering potential benefits in terms of reduced surgical trauma and enhanced control during dissection.

While our perioperative management strategy was successful, further studies are needed to establish standardized protocols for managing patients with hereditary AT deficiency undergoing thoracic surgery. The experience described in this case may provide valuable insight for clinicians encountering similar cases and contribute to the development of more effective and safer surgical strategies for patients with coagulation disorders.

## SUPPLEMENTARY MATERIALS

Supplementary Video 1 This video demonstrates the key steps of the uniportal robotic-assisted thoracic surgery (U-RATS), including port placement, instrument manipulation, and lesion resection. The video duration is approximately 2 minutes and 40 seconds.
The procedure was performed using the U-RATS technique. The patient was positioned in the supine position under general anesthesia. A 4.0-cm skin incision was made at the right 5th intercostal space along the midaxillary line to access the thoracic cavity. The Da Vinci Xi (Intuitive Surgical, Sunnyvale, California, USA) robot was positioned, and Arm 4 was moved caudally. An 8-mm, 30° oblique endoscope was attached to Arm 1, a fenestrated bipolar forceps to Arm 2, and a long bipolar grasper to Arm 3. The ports were placed within the wound in the order of Arm 1, Arm 2, and Arm 3 from the dorsal side. The depths of the ports were adjusted to prevent interference (**Fig. 3A**). The lesion, identified as a soft induration with slight elevation in the right anterior mediastinum, was excised via partial thymectomy. The cystic lesion was resected with a maximum diameter of 3.2 cm. Console time was 32 minutes, total operative time was 1 hour 6 minutes, and there was no blood loss.

## DECLARATIONS

### Availability of data and materials

Data sharing is not applicable because no datasets were generated or analyzed in the current study.
